# Growth and physiological responses of isohydric and anisohydric poplars to drought

**DOI:** 10.1093/jxb/erv195

**Published:** 2015-05-07

**Authors:** Ziv Attia, Jean-Christophe Domec, Ram Oren, Danielle A. Way, Menachem Moshelion

**Affiliations:** ^1^Institute of Plant Sciences and Genetics in Agriculture, The Robert H. Smith Faculty of Agriculture, Food and Environment, The Hebrew University of Jerusalem, Rehovot 76100, Israel; ^2^Bordeaux Sciences Agro UMR INRA-ISPA 1391, 33195, Gradignan, France; ^3^Department of Forestry and Environmental Resources, North Carolina State University, Raleigh, NC 27695, USA; ^4^Nicholas School of the Environment and Earth Sciences, Duke University, Durham, North Carolina 27708, USA; ^5^Department of Forest Ecology & Management, Swedish University of Agricultural Sciences (SLU), SE-901 83, Umeå, Sweden; ^6^Department of Biology, University of Western Ontario, London, ON, N6A 5B7, Canada

**Keywords:** Bioenergy, biomass, carbon, hydraulic conductance, stomata, transpiration

## Abstract

Understanding how different plants prioritize carbon gain and drought vulnerability under a variable water supply is important for predicting which trees will maximize woody biomass production under different environmental conditions. Here, *Populus balsamifera* (BS, isohydric genotype), *P. simonii* (SI, previously uncharacterized stomatal behaviour), and their cross, *P. balsamifera x simonii* (BSxSI, anisohydric genotype) were studied to assess the physiological basis for biomass accumulation and water-use efficiency across a range of water availabilities. Under ample water, whole plant stomatal conductance (g_s_), transpiration (E), and growth rates were higher in anisohydric genotypes (SI and BSxSI) than in isohydric poplars (BS). Under drought, all genotypes regulated the leaf to stem water potential gradient via changes in g_s_, synchronizing leaf hydraulic conductance (K_leaf_) and E: isohydric plants reduced K_leaf_, g_s_, and E, whereas anisohydric genotypes maintained high K_leaf_ and E, which reduced both leaf and stem water potentials. Nevertheless, SI poplars reduced their plant hydraulic conductance (K_plant_) during water stress and, unlike, BSxSI plants, recovered rapidly from drought. Low g_s_ of the isohydric BS under drought reduced CO_2_ assimilation rates and biomass potential under moderate water stress. While anisohydric genotypes had the fastest growth under ample water and higher photosynthetic rates under increasing water stress, isohydric poplars had higher water-use efficiency. Overall, the results indicate three strategies for how closely related biomass species deal with water stress: survival-isohydric (BS), sensitive-anisohydric (BSxSI), and resilience-anisohydric (SI). Implications for woody biomass growth, water-use efficiency, and survival under variable environmental conditions are discussed.

## Highlight

Isohydric poplars have high water-use efficiency, while anisohydric poplars show faster growth under a variable water supply, with implications for performance of the different genotypes for woody biomass production.

## Introduction

Society’s dependence on fossil fuels contributes to greenhouse gas emissions and environmental pollution, leading to a demand for renewable energy sources. Woody biomass represents a renewable resource with multiple industrial applications that can serve feedstock needs for the cellulosic energy and biofuels industry without conflicting with food production (Kenney *et al.*, 1990), and trees are expected to account for 377 million dry tons of the 1.37 billion dry tons total biomass necessary for a 30% replacement of US petroleum consumption with biofuels by 2030 (Perlack *et al.*, 2005). Thus, tree growth rate, which underlies dry biomass gain, is a fundamental characteristic that can be used to increase productivity in tree plantations. As hybrid poplars are among the fastest growing temperate trees in the world, they serve as a promising feedstock for biofuels and other value-added products (Sannigrahi *et al.*, 2010). Significant efforts have therefore been invested in poplar research, including genome sequencing (Tuskan *et al.*, 2006; Bolger *et al.*, 2014), in an attempt to produce high-yield cultivars.

Of the environmental factors constraining tree growth rate, water is usually the most critical, and water stress restricts plant growth and yield (Bréda *et al.*, 2006; McDowell *et al.*, 2008). This is at least partly because water loss via transpiration (E) is an inevitable consequence of photosynthesis, via the link between CO_2_ diffusion into, and water flux out of, stomata (von Caemmerer and Baker, 2007). Stomatal conductance (g_s_) thereby acts as a key control on both tree water loss and carbon gain, while carbon gain is closely linked to biomass production. At the leaf level, the ratio between CO_2_ uptake and E (i.e. leaf-level water-use efficiency, WUE_l_) is ~3–40 µmol CO_2_ mmol H_2_O^-1^ across different well-watered poplar genotypes (Liang *et al.*, 2006; Soolanayakanahally *et al.*, 2009; Larchevêque *et al.*, 2011), implying a more than 10-fold difference in potential carbon assimilation under a variety of soil water and evaporative demand conditions. These variations in WUE_l_ often result mainly from variations in g_s_ and not differences in net CO_2_ assimilation rate (A_N_) or photosynthetic capacity (Blum, 2005); therefore, an increase in WUE_l_ usually results in reduced photosynthesis and yield (Flexas *et al.*, 2013). Despite decades of research on stomatal physiology, the complex mechanisms that adjust stomatal aperture and regulate g_s_ are still poorly understood although they are vital for plant function, especially when water supply is limited (Tardieu and Simonneau, 1998). Nevertheless, there seems to be general agreement that stomata sense leaf water potential (Ψ_leaf_) so that both g_s_ and leaf hydraulic function decline when Ψ_leaf_ decreases (Brodribb and Cochard, 2009; Domec *et al.*, 2009).

Leaf and plant water transport capacity can be quantified in terms of leaf and whole plant hydraulic conductance (K_leaf_ and K_plant_, respectively). Plants with high hydraulic conductance can supply water rapidly from their roots to the leaves, maximizing g_s_, A_N_, and, ultimately, productivity under well-watered conditions (Nardini and Salleo, 2000). Indeed, differences in xylem traits, such as vessel diameter and sapwood area, as well as the water potential gradient from the soil/root to the leaf may generate variation in E and g_s_ among tree species, as well as within a species (Kleiner *et al.*, 1992; Vivin *et al.*, 1993; Comas *et al.*, 2002; Wikberg and Ögren, 2004; Cocozza *et al.*, 2010). However, when evaporative demand exceeds the ability to supply water to the transpiration stream, g_s_ declines to protect the plant hydraulic system from cavitation (Zimmermann, 1983; Oren *et al.*, 1999; Sperry, 2000). Consistent with earlier work documenting the coordination of g_s_ with K_leaf_ (Meinzer *et al.*, 1995), recent work has revealed that maximum g_s_ is very sensitive to K_leaf_ (Ewers *et al.*, 2000; Brodribb and Holbrook, 2003; Woodruff *et al.*, 2007; Domec *et al.*, 2009). As K_leaf_ declines, owing to cavitation or regulated changes in mesophyll conductance (Johnson *et al.*, 2009), Ψ_leaf_ will also decline, stomata will close, and yield will be negatively affected (Sack and Holbrook, 2006). Therefore, maintaining the integrity of the root to leaf water continuum while avoiding embolism during transpiration is essential for sustaining photosynthetic gas exchange and growth in plants (Meinzer and McCulloh, 2013).

Depending on their genetically dictated molecular and physiological attributes, plants budget their water in very different ways, along a continuum that ranges from the water-conserving behaviour displayed by isohydric plants to the ‘risk-taking’ behaviour displayed by anisohydric plants (Tardieu and Simonneau, 1998; Moshelion *et al.*, 2014; Sade and Moshelion, 2014). In isohydric species, stomata conservatively regulate plant water status by controlling the rate of water loss to the atmosphere such that it matches the capacity of the soil–plant hydraulic system to supply water to leaves. In order to decrease the risk of hydraulic dysfunction and leaf dehydration, isohydric plants maintain a constant, or nearly constant, minimum daily Ψ_leaf_ (thus reflecting a narrowing soil–leaf water potential gradient) and relative water content by reducing g_s_ and E under water stress. Anisohydric plants, on the other hand, allow Ψ_leaf_ to decrease under drought conditions relative to a well-watered environment, thus reaching a lower Ψ_leaf_ and relative water content with rising evaporative demand and maintaining the driving force for water flow to leaves (reviewed by Moshelion *et al.*, 2014). Yet, the physiological mechanism for the regulation of isohydric and anisohydric behaviours is not fully understood (Klein, 2014; Martínez‐Vilalta et al., 2014).

These different stomatal behaviours have implications for selecting the appropriate tree species or genotype to maximize yield and biomass production for bioenergy. Because isohydric plants are expected to reduce g_s_ as soil water becomes limiting, water loss and growth rates should also be reduced. Consequently, higher WUE_l_ can be expected in isohydric plants than in anisohydric plants as soil dries, implying that isohydric trees would not maximize yield on a plantation, though they may be the most water-use efficient trees for producing biomass. By contrast, under prolonged severe drought, isohydric trees might be expected to survive, whereas anisohydric trees are expected to die, generating no yield at all (reviewed by Moshelion *et al.*, 2014). Although hybrid poplars are generally considered to be relatively isohydric (Tardieu and Simonneau, 1998), they actually vary widely in their stomatal sensitivity to soil and air drying and susceptibility to xylem cavitation (Arango-Velez *et al.*, 2011; Silim *et al.*, 2009).

In this work, three poplar genotypes were assessed to determine how fundamental differences in stomatal behaviour, photosynthesis, leaf hydraulics, and leaf size affect growth, drought tolerance, water-use efficiency, and drought recovery rate with the goal of identifying stomatal strategies to maximize biomass productivity under variable water supplies. The genotypes used were *Populus balsamifera* L. (BS), a North American boreal species that maintains a constant Ψ_leaf_ under mild water stress (Almeida-Rodriguez *et al.*, 2010), and is considered isohydric; *Populus simonii* carr. (SI), a fast-growing Asian species that has unknown stomatal regulation behaviour; and their cross, *P. balsamifera x simonii* (BSxSI), which has been reported to act in an anisohydric manner (Almeida-Rodriguez *et al.*, 2010). Previous studies reported physiological differences in A_N_, g_s_, and WUE_l_, and molecular differences (e.g. aquaporin expression) between BS and BSxSI exposed to a range of water stress (Soolanayakanahally *et al.*, 2009; Almeida-Rodriguez *et al.*, 2010), despite their close genetic relationship. It was hypothesized that isohydric poplars would produce less biomass but have greater WUE_l_, and therefore would have greater survival and recovery, than anisohydric plants in response to prolonged water deprivation. By contrast, anisohydric poplars would accumulate more biomass under no water stress, as well as mild to moderate drought stress, owing to the maintenance of high g_s_ and A_N_ under low Ψ_leaf_, but would have low survival rates during prolonged drought. It was also hypothesized that the reduction of Ψ_leaf_ in anisohydric plants would result from the coordinated maintenance of high K_leaf_ and high g_s_ as water stress progressed.

## Methods

Complementary experiments were conducted in two locations, Duke University and the Hebrew University of Jerusalem, using a single set of cuttings from dormant stems of the three poplar genotypes (supplied by Agriculture and Agri-Food Canada, Ottawa, ON, Canada) that were split between the two locations.

### Duke University, NC, USA

Stem cuttings were put into 3.9L pots filled with Fafard 52 mix potting soil (www.sungro.com; Agawam, MA, USA) and grown in the Duke University Phytotron greenhouses for ~4 months to root and establish leaves. Plants were then moved to fully controlled conditions [25/20°C day/night, 18/6h light/dark, 70% relative humidity (RH), and 700 µmol photons m^-2^ s^-1^] in growth chambers (Environmental Growth Chambers, Chagrin Falls, OH, USA), and were watered as needed to maintain a moist growing medium and fertilized with half-strength Hoagland’s solution once per week. Gravimetric soil water content (SWC_g_) was calculated as the ratio of the mass of water in the soil sample to the mass of dry soil.

The growth rate was measured on well-watered trees grown in 0.3L pots maintained in these growth chambers. Cuttings with one lateral bud were grown for 62–65 days (until they were 30–40cm tall) and then cut at the soil surface and weighed for fresh weight. The mass of each shoot was normalized to its stem diameter (mean of three measurements taken at the soil surface level with digital callipers). Leaf size and total leaf area per plant were measured on the same trees using a leaf area meter (Li-3100; LI-COR, Lincoln, NE, USA). Stomatal density measurements were made on a subset of leaves using a rapid imprinting technique (Geisler and Sack, 2002), which allowed the reliable scoring of hundreds of stomata at the same time. In brief, light-bodied vinyl polysiloxane dental resin (Heraeus-Kulzer, http://heraeus-dental.com) was attached to the abaxial and adaxial leaf sides and then removed as soon as it had dried (1min). The resin epidermal imprints were covered with transparent nail polish, which was removed once it had dried. The nail-polish imprints were put on microscope slides and photographed under a bright-field inverted microscope (Zeiss Axio Imager, http://www.zeiss.com) with a QImaging MicroPublisher 5.0 MP colour camera (http://www.qimaging.com). Stomatal images were analysed using IMAGEJ (http://rsb.info.nih.gov/ij).

Photosynthetic capacity was assessed on five well-watered individuals per genotype with a portable photosynthesis system (Li-6400; LI-COR). Responses of A_N_ to changes in intercellular CO_2_ concentrations were made at a leaf temperature of 25°C, a vapor pressure deficit of ~1.6 kPa, and saturating light (1500 µmol m^-2^ s^-1^ photosynthetic photon flux density); ambient cuvette CO_2_ concentrations were lowered stepwise from 400 to 50 µmol mol^-1^, returned to 400 µmol mol^-1^, and subsequently raised stepwise to 2000 µmol mol^-1^. Both maximum Rubisco carboxylation rates (V_cmax_) and maximum electron transport rates (J_max_) values were calculated according to Farquhar *et al.* (1980), using Rubisco kinetic parameters from von Caemmerer *et al.* (1994).

When the plants were ~1 m tall, they were moved to a semi-controlled greenhouse (18/6h light/dark, 50–60% RH, 25°C and natural irradiance). Measurements took place from June to August 2013. The plants were exposed to progressive reductions in SWC_g_ encompassing three categories of water stress based on the lowest SWC_g_ measured in most plants (~30% SWC_g_) and on SWC_g_ at field capacity (>70% SWC_g_): 70–100% SWC_g_, 50–69% SWC_g_, and 30–49% SWC_g_. Note that the values in the high SWC_g_ class were always >70% but remained mostly <85%. The SWC_g_ was continuously measured with Theta Probes (model ML2x; Delta-T Devices Ltd, Cambridge, UK) connected to a CR10 data logger (Campbell Scientific, Inc., Logan, UT, USA). These SWC_g_ values were then used to calculate Ψ_soil_ based on a ‘dynamic’ water retention function, obtained by pairing the values of water content and a water pressure head placed in the pot at a given time (Klute, 2003).

Unless mentioned otherwise, all measurements during the dry-down experiment were taken from randomly selected individuals from each genotype. The drought treatments at Duke lasted 5 days maximum for each plant, and involved 42 BS plants, 121 BSxSI plants, and 43 SI plants. For a selected plant, point measurements of g_s_, E, and A_N_ were made with a portable photosynthesis system (Li-6400; LI-COR) between 09:00 and 12:00 hours, with the cuvette set to growth conditions in the greenhouse. Immediately after, Ψ_leaf_ was assessed with a Scholander pressure chamber (PMS Instruments, Albany, OR, USA) and the stem water potential (Ψ_stem_) was estimated using the bagged-sealed leaf technique (Begg and Turner, 1970). These values were used to estimate K_leaf_ and K_plant_ as described in Equations 1 and 2:

$${\text{K}}_{\text{leaf}}=\text{ E}/\left(\Psi_{\text{stem}}-\;\Psi_{\text{leaf}}\right)$$ (1)

$${\text{K}}_{\text{plant}}=\text{ E}/\left(\Psi_{\text{soil}}-\;\Psi_{\text{leaf}}\right)$$ (2)

### Hebrew University of Jerusalem, Rehovot, Israel

A similar set of cuttings from the same three poplar genotypes were grown in Israel to determine whole plant water use and responses to drought. Whole plant daily transpiration (DT) and growth were determined using an array of lysimeters placed in a greenhouse at the Faculty of Agriculture, Rehovot, Israel, as described in detail previously (Sade *et al.*, 2009). Briefly, cuttings were planted in 3.9L pots filled with potting soil and grown under semi-controlled conditions of 30/25°C day/night under natural day length and light in Rehovot, Israel, from April to May 2014, with ample water supply. Each pot was placed on a temperature-compensated load cell with a digital output (Vishay Tedea-Huntleigh, Netanya, Israel), and was sealed to prevent evaporation from the soil surface. The weight output of the load cells was monitored every 15 s and the average readings over 3min periods were logged (Campbell Scientific CR1000 Data Logger) for further analysis. DT was assessed as the difference in mass between 04:30 and 18:00 hours.

A drainage hole at the base of the lysimeters maintained a constant water level following irrigation events, enabling the calculation of the weight gained by the plant (ΔPW_k_) between two consecutive irrigation events as described in Equation 3:

$$\Delta {\text{PW}}_{\text{k}}={\text{ W}}_{\text{k }+\text{ 1}}-{\text{ W}}_{\text{k}}$$ (3)

where W_k_ and W_k+1_ are the total weight of the pot placed on the lysimeter on two consecutive days (days k and k+1). Therefore, the plant weight gain over the entire experiment period was the sum of the daily plant weight gains from the first to the last day. Agricultural water use efficiency (WUE_a_) was calculated as the cumulative weight gain over cumulative transpiration during measurements days as describe in Equation 4:

$${\text{WUE}}_{\text{a}}=\;\sum \left(\Delta {\text{PW}}_{\text{k}}\right)/\sum \text{DT}$$ (4)

The plants were watered daily until the onset of drought treatment, where no watering was applied until the SWC_g_ in each pot fell below 30%. Because the trees growing in Israel were smaller than those at Duke, the drought varied between 10 and 20 days in duration (depending on plant size); the number of individuals from each genotype varied based on initial availability, and survival during growth and drought (four from BS, 20 from BSxSI, 15 from SI). Once SWC_g_ fell below 30%, watering was reinitiated. Recovery patterns from severe drought stress (SWC_g_ <30%) were determined as the proportion of DT after the return of irrigation to each plant compared to maximal DT prior to drought treatment.

### Statistical analyses

Shapiro–Wilk tests for normal distribution of the data were made prior to Tukey’s honestly significant difference (HSD) tests used for comparisons of means; comparison to controls were made using the Dunnett’s method. Both tests were considered to be significantly different at *P* < 0.05; all statistics were analysed with JMP 10 Pro (SAS Institute Inc., Cary, NC, USA).

## Results

**Fig. 1.**
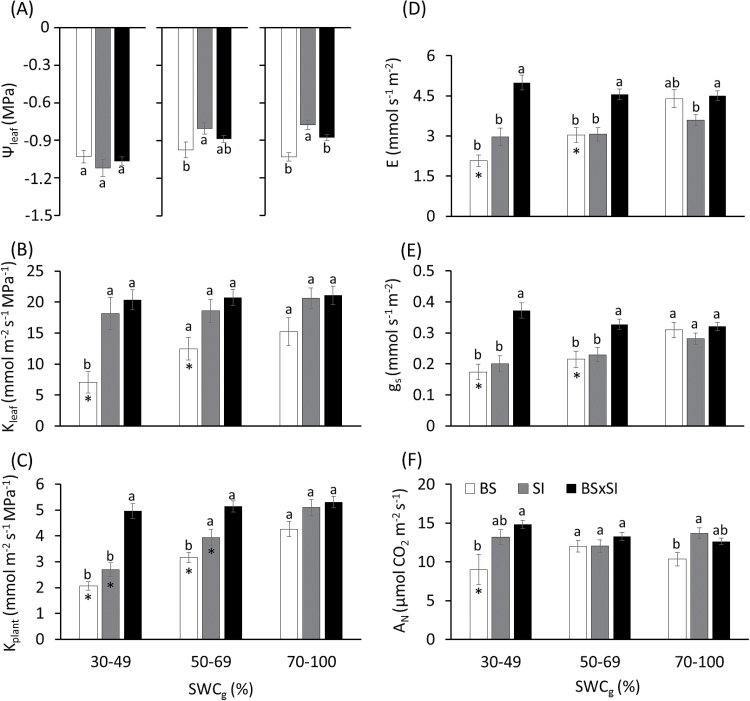
Mean differences in (A) Ψ_leaf_, (B) K_leaf_, (C) K_plant_, (D) E, (E) g_s_, and (F) A_N_ in three poplar genotypes under three SWC_g_ treatments grown in a semi-controlled greenhouse. Data are shown as means ± SE (BS; n = 42), (SI; n = 210), and BSxSI (n = 311). Different letters above the columns indicate significant differences between the three poplar genotypes within an SWC_g_ bin according to Tukey’s HSD test, *P* < 0.05. Asterisks indicate significant differences in comparisons within a genotype to well-irrigated controls using Dunnett’s method, *P* < 0.05.

**Fig. 2.**
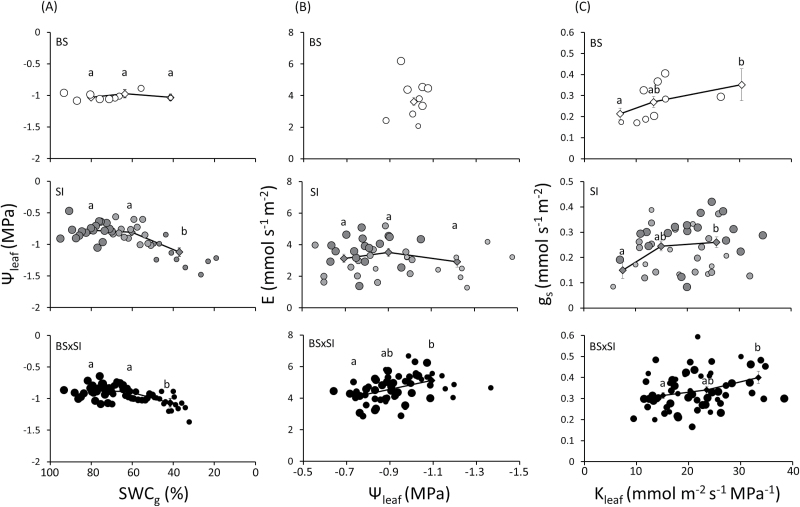
Relationship between (A) SWC_g_ and Ψ_leaf_, (B) Ψ_leaf_ and E, and (C) K_leaf_ and g_s_ in three poplar genotypes grown in a semi-controlled greenhouse. Data binned by SWC_g_, every circle is a 5 point average: large circles, 70–100% SWC_g_, medium circles, 50–69% SWC_g_, small circles, 30–49% SWC_g_. BS (n = 42), SI (n = 210), BSxSI (n = 311). Lines connect the mean ± SE of the SWC_g_ bins (30–49%, 50–69%, 70–100%). Different letters above the SE bars indicate significant differences between means using Tukey’s HSD test, *P* < 0.05.

Under well-watered conditions (70–100% SWC_g_), Ψ_leaf_ of the SI was significantly less negative than the BS and BSxSI, but Ψ_leaf_ differences between the three genotypes disappeared at 30–49% SWC_g_ (Fig. 1A). Thus, only the BS presented isohydric behaviour, maintaining constant Ψ_leaf_ with declining SWC_g_ (Fig. 2A), and the stem water potential (Ψ_stem_) showed the same tendency (Supplementary Fig. 1A). As a consequence, the water potential difference between the stem and the leaf (∆Ψ_leaf_) did not vary between the three genotypes: ∆Ψ_leaf_ remained constant as SWC_g_ decreased, generating a constant driving force (of around 0.3MPa) for water ﬂow from the stem to the leaf (Supplementary Fig. 1B). This behaviour was made ​​possible by the fact that the BS sharply reduced E and g_s_ in response to the declining SWC_g_, while BSxSI and SI kept higher E and g_s_ as water depletion progressed, and were thus insensitive to the declining Ψ_leaf_ (Figs 1D,E and 2A,B).

K_leaf_ showed a similar pattern to E and g_s_; while SI and BSxSI kept K_leaf_ relatively constant as SWC_g_ declined, BS K_leaf_ decreased by ~50% under the same conditions (Fig. 1B). Thus, g_s_ declined in concert with the decrease in K_leaf_ in the isohydric BS genotype, but g_s_ was less tightly correlated with decreases in K_leaf_ in the two anisohydric poplars (Fig. 2C). Only the BSxSI maintained constant K_plant_ as SWC_g_ decreased (Fig. 1C).

There were no significant differences in the photosynthetic capacity between the genotypes, as measured by maximum V_cmax_ J_max_ (means ± SE: V_cmax_ = 125.7±5.2 µmol CO_2_ m^-2^ s^-1^; J_max_ = 167.8±8.6 µmol CO_2_ m^-2^ s^-1^). However, instantaneous measurements of A_N_ in the greenhouse revealed that the SI had a higher A_N_ at 70–100% and the BS had the lowest A_N_ at 30–49% SWC_g_ (Fig. 1F).

**Fig. 3.**
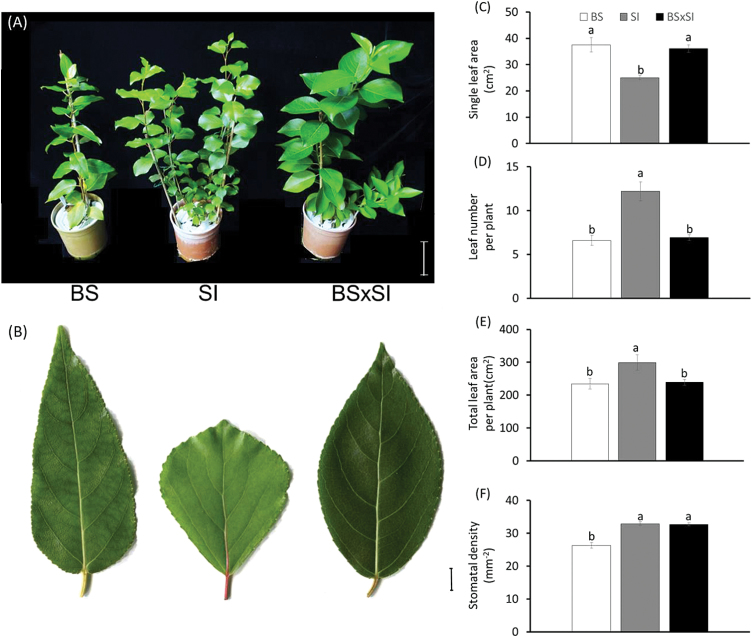
Canopy and leaf morphology characteristics of the three poplars genotypes: (A) image of representative 5-month-old seedlings grown in a semi-controlled greenhouse and used for the experiments (bar = 10cm); (B) representative fully mature, expanded leaves (bar = 1cm); (C) single leaf area; (D) leaf number per plant; (E) total canopy area per plant; and (F) stomatal density in a 1mm^2^ sample area. Data are shown as means ± SE; BS (n = 14), SI (n = 18), BSxSI (n = 34). Different letters above the columns indicate significant differences between treatments using Tukey’s HSD test, *P* < 0.05 (this figure is available in colour at JXB online).

**Fig. 4.**
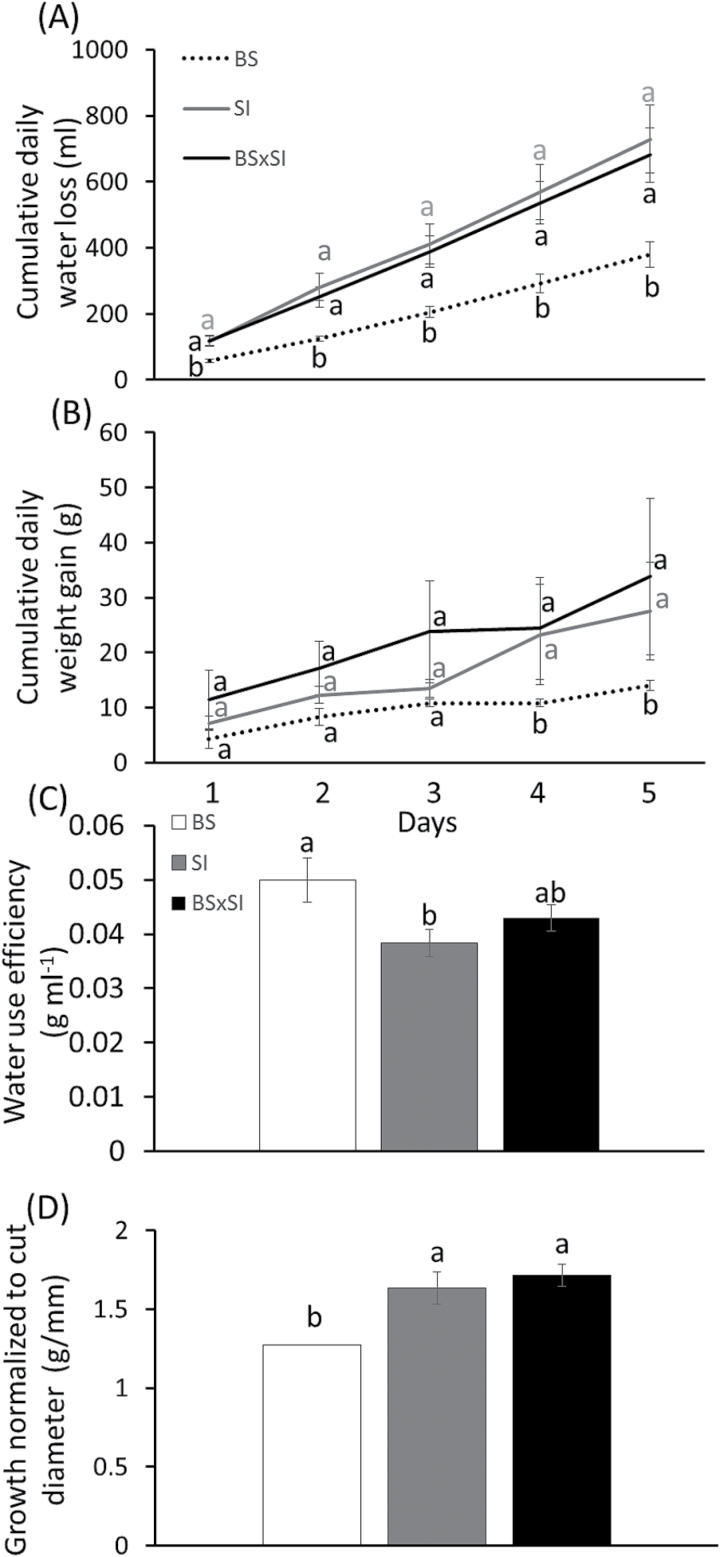
(A) Whole-plant cumulative water loss, (B) cumulative plant weight gain, (C) agricultural WUE (cumulative transpiration to cumulative weight gain ratio), and (D) growth normalized to cut diameter (g fresh mass/mm) in three poplar genotypes grown under well-irrigated conditions in a semi-controlled greenhouse. Data are shown as means ± SE: for (A-C), BS (n = 4), SI (n = 15), and BSxSI (n = 19); for (D) BS (n = 5), SI (n = 6), and BSxSI (n = 16). White, BS; grey, SI; black, BSxSI. Different letters above the columns indicate significant differences between the genotypes for each day, according to Tukey’s HSD test, *P* < 0.05.

The different genotypes varied in leaf size and number (Fig. 3A,B). BS and BSxSI had bigger leaves than SI seedlings (Fig 3B,C), but SI plants had more leaves per plant, such that there was a larger total leaf area in SI plants than in the other two genotypes (Fig 3D,E). Owing to its lower total leaf area and stomatal density (Fig. 3F), BS plants had the lowest stomatal number per plant (i.e. the gas exchange capacity per plant), while SI seedlings had the highest. Under well-watered conditions, the BS genotype also had significantly lower growth rates than the two anisohydric genotypes (Figure 4D).

**Fig. 5.**
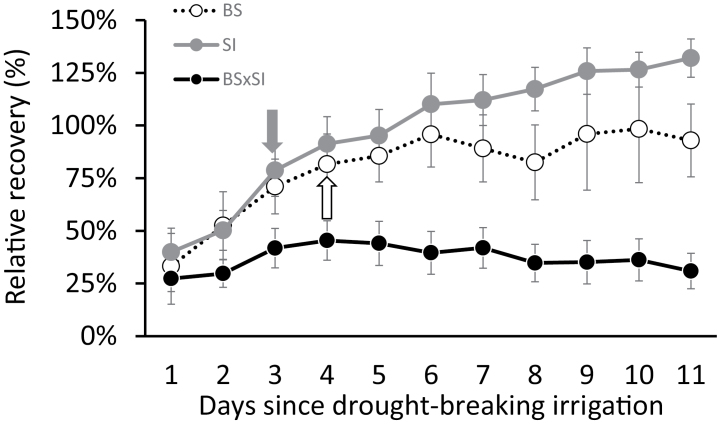
Recovery rate (relative to pre-treatment transpiration level) from water deprivation of ~30% SWC_g_. Data are shown as means ± SE: BS (n = 4), SI (n = 15), and the BSxSI (n = 19). Arrows (with colours matching the symbols for the respective genotypes) indicate when post-stress transpiration rates were not significantly different from the pre-treatment values according to a Student *t*-test, *P* < 0.05.

To better understand how these morphological and physiological differences contribute to plant growth rates, water-use efficiency, and drought tolerance, whole-plant transpiration, growth rate, and WUE_a_ were measured. The SI and BSxSI had higher cumulative transpiration and weight gain than the BS under well-watered conditions (Fig. 4A,B,D), which did not correspond with a higher leaf-level E and g_s_ (Fig. 1D,E), but could be explained by considering the different canopy morphology and stomatal densities between the genotypes (Fig. 3). However, the isohydric BS gained more biomass for a given amount of water transpired, generating a higher WUE_a_ compared to the anisohydric SI plants (Fig 4C). The recovery patterns from severe water stress (reaching SWC_g_ <30%) showed that the BS and SI fully recovered within 3–4 days of irrigation, while the BSxSI did not recover to their initial transpiration rates even after 11 days of irrigation (Fig. 5).

## Discussion

**Fig. 6.**
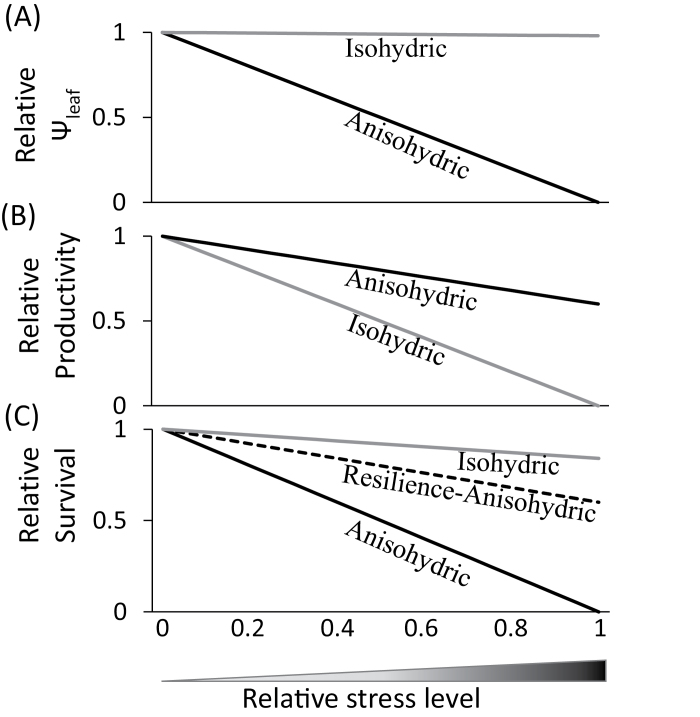
Conceptual model for behaviour of isohydric versus anisohydric plants by means of regulating (A) Ψ_leaf_, (B) productivity, and (C) survival in response to increasing relative water stress, which accounts for changes in the SWC_g_ and the period of time water stress was applied (this figure is available in colour at JXB online).

This study demonstrates that three genotypes of poplar, a key woody biomass species, have different strategies to cope with drought stress, with implications for their suitability for biomass production. The contrasting stomatal and leaf hydraulic behaviours between genotypes ranged from a rapidly responding isohydric behaviour (BS), which is hypothesized to increase survival under drought at the cost of low biomass production, to an anisohydric behaviour (SI and BSxSI) that is thought to allow carbon uptake and maintain high growth rates for a longer period during drought, but to expose the plant to greater risk of drought-induced mortality if the drought persists. Overall, the results indicated three strategies for how the closely related biomass genotypes deal with water stress: survival-isohydric, sensitive-anisohydric, and resilience-anisohydric (Fig. 6). By reducing hydraulic and stomatal conductance to maintain a constant Ψ_leaf_, the isohydric poplars minimized the exposure of their leaves to water stress, but also decreased their ability to fix carbon for growth as the soil dried. By contrast, the anisohydric plants kept a high A_N_ while Ψ_leaf_ declined, which should enable higher productivity in the anisohydric poplars, but also made them more vulnerable to damage from prolonged drought stress (Figs 2, 4, 5, 6). Given that recent work in 37 hybrid and pure species of poplar has shown that mean water potentials at which 50% of conductivity is lost range between −1.3 and −1.5MPa, with a large number of hybrids losing 50% of conductivity at water potentials >−1MPa (Fichot *et al.*, 2015), the Ψ_stem_ values of near −0.9MPa shown here were likely sufficient to induce significant cavitation.

While the ability of the isohydric BS to maintain high Ψ_leaf_ and Ψ_stem_ in both well-irrigated and drought conditions compared to the anisohydric BSxSI (Fig. 1A, Supplementary Fig. 1A) has already been documented (Almeida-Rodriguez *et al.*, 2010), the stomatal strategy of the SI has not. Here, the paternal SI demonstrated the opposite water balance regulation strategy to the maternal BS used to generate the crosses. The conservative water balance regulation of BS comes at the cost of slower growth rates (Figs 1F and 4B,D), which should be exacerbated by a fast reduction in E, g_s_, K_leaf_, and A_N_ as soil dries, as hypothesized. Yet, the BS plants benefitted from this behaviour through their ability to maintain a high Ψ_leaf_ under dry soils and, if subjected to drought, BS plants recovered faster than the anisohydric BSxSI cross (Fig. 5), and will thus likely have higher survival in dry conditions. By contrast, the anisohydric behaviour of the SI and BSxSI plants enabled them to sustain faster growth rates (Fig. 4B,D) through longer periods of high E and g_s_ as water availability declined (Supplementary Fig. 1A,B, Fig. 4B,D). This, in turn, enabled longer periods of high A_N_ as SWC_g_ decreased (Fig. 1F), making these poplars more suited for high biomass productivity. In fact, this anisohydric behaviour was suggested to be an agronomic trait, because anisohydric plants may outperform isohydric plants in terms of growth and yield (McDowell *et al.*, 2008; Sade *et al.*, 2009; Sade *et al.*, 2012). As hypothesized, the reduction in Ψ_leaf_ of the SI and BSxSI plants was possible by maintaining high K_leaf_ (Fig. 1B). Nevertheless, the risk of keeping high hydraulic conductance as well as high g_s_ during deteriorating SWC_g_ might be hydraulic failure. Thus, under short-term stress conditions, the cost of anisohydry should be slow drought recovery (as seen in the BSxSI), and if the stress is prolonged, possible tree mortality.

Interestingly, despite the fact that the SI and BSxSI presented very similar anisohydric stomatal regulation (and therefore similar growth patterns), SI plants showed much better recovery from drought compared with the BSxSI plants (Fig. 5). With all else being equal, leaf and whole-plant tolerance to low SWC_g_ should be conferred by the ability to provide transport pathways from major veins to the sub-stomatal cavity, which is generally associated with small leaf size (McKown *et al.*, 2010; Scoffoni *et al.*, 2011), and big leaved plants are less adapted to dry habitats (Gibson, 1998; Ackerly, 2004). Therefore, the greater drought recovery ability of the SI may be partly due to its smaller leaves, although other parameters, such as its ability to maintain high K_leaf_ while reducing its K_plant_ during drought, might also serve as embolism defence mechanisms (Domec and Johnson, 2012). The fact that BSxSI poplars maintained high K_leaf_ and K_plant_ (but suffered from slow water-stress recovery), while both K_leaf_ and K_plant_ were reduced in BS poplars (Fig. 1B,C), supports this hypothesis. In addition, the ability of the SI to maintain relatively low E but relatively high A_N_ for longer periods under drought, together with the larger SI leaf area per plant, provides additional advantages for growth under drought.

Poplars are long-lived trees characterized by a dioecious breeding system, wind dispersal of pollen and seeds, clonality, and often continental-scale distribution; as a result, poplars potentially comprise interbreeding populations of immense size. These life history traits typify a plant expected to exhibit abundant genetic variation (Breen *et al.*, 2009), and hybrid poplars are a promising feedstock for multiple industrial applications (Sannigrahi *et al.*, 2010) owing to their large germplasm and their status as the model species for tree genomics (Tuskan *et al.*, 2006). In the long developmental process of breeding new tree genotypes, understanding the physiological responses of the plants to their intended growth environment is a crucial step to developing appropriate cultivars for commercial plantations. Under high soil moisture, anisohydric poplars (such as the SI and BSxSI) had a clear advantage because of their faster growth and higher photosynthetic rates, which may facilitate higher biomass production. Yet increasing demand for food and energy, combined with rising pressure for land conversion, may affect productivity (Harvey and Pilgrim, 2011) as biomass crops are grown on increasingly marginal lands to prevent competition with food production (Murphy *et al.*, 2011; Swinton *et al.*, 2011). Under these conditions, planting the isohydric BS is preferable, because it has high water-use efficiency and is able to grow and survive under poorer conditions, although its performance is limited in terms of growth.

While the implications of varied stomatal regulation strategies for growth, water-use efficiency, and survival under a variable environment should be tested in the field, the SI’s dynamic resilience-anisohydric behaviour patterns (Fig. 6) might be the ultimate strategy for growing under mild to moderate drought conditions, and may provide a suitable role model for future development in woody biomass production.

## Supplementary data

Figure S1: The effect of SWC_g_ on (A) Ψ_leaf_ and (B) the difference between Ψ_stem_ and Ψ_leaf_ of three poplar genotypes. Data is shown as means ± SE from at least 20 independent measuring days and 24 technical repetitions per day. Different letters above the columns indicate significant differences between treatments according to Tukey’s HSD test, *P* < 0.05. Asterisks indicate significant differences within a genotype in comparisons to well-irrigated controls using Dunnett’s method, *P* < 0.05.

Supplementary Data

## Abbreviations:

A_N_: net CO_2_ assimilation rate
DT: whole plant daily transpiration
E: transpiration rate
g_s_: stomatal conductance
J_max_: maximum electron transport rates
K_leaf_: leaf hydraulic conductance
K_plant_: plant hydraulic conductance
Ψ_leaf_: leaf water potential
Ψ_soil_: soil water potential
Ψ_stem_: stem water potential
SWC_g_: gravimetric soil water content
V_cmax_: maximum Rubisco carboxylation rates
W_k_: pot weight on day k
WUE_a_: agronomic water use efficiency
WUE_l_: leaf-level water-use efficiency.
