# Supplementary material for: Growth and physiological responses of isohydric and anisohydric poplars to drought
Source: J Exp Bot. 2015 May 7;66(14):4373–81. doi: 10.1093/jxb/erv195 (PMC4493787; doi:10.1093/jxb/erv195)
Supplement: Supplementary Data [file supp_66_14_4373__index.html]

Growth and physiological responses of isohydric and anisohydric poplars to drought — Growth and physiological responses of isohydric and anisohydric poplars to drought — Supplementary Data 

# Growth and physiological responses of isohydric and anisohydric poplars to drought

## Supplementary Data

Data files

**Files in this Data Supplement:**

- Supplementary Data - Supplementary Data
