# Supplementary material for: Growth and physiological responses of isohydric and anisohydric poplars to drought
Source: J Exp Bot. 2015 May 7;66(14):4373–81. doi: 10.1093/jxb/erv195 (PMC4493787; doi:10.1093/jxb/erv195)
Supplement: Supplementary Data [file supp_erv195_erv195_144774_supplementary_data.pdf]

Growth and Physiological Responses of Isohydric and Anisohydric Poplars to Drought

Ziv Attia, Jean-Christophe Domec, Ram Oren, Danielle A. Way and Menachem Moshelion

Supplementary Material

Figure S1: The effect of SWC_g on (A) Ψ_{leaf}; and (B) the difference between Ψ_{stem} and Ψ_{leaf} of three poplar genotypes. Data is shown as means ± SE from at least twenty independent measuring days and 24 technical repetitions per day. Different letters above the columns indicate significant differences between treatments according to Tukey's HSB test, $P < 0.05$. Asterisks indicate significant differences within a genotype in comparisons to well irrigated controls using Dunnett's method, $P < 0.05$.

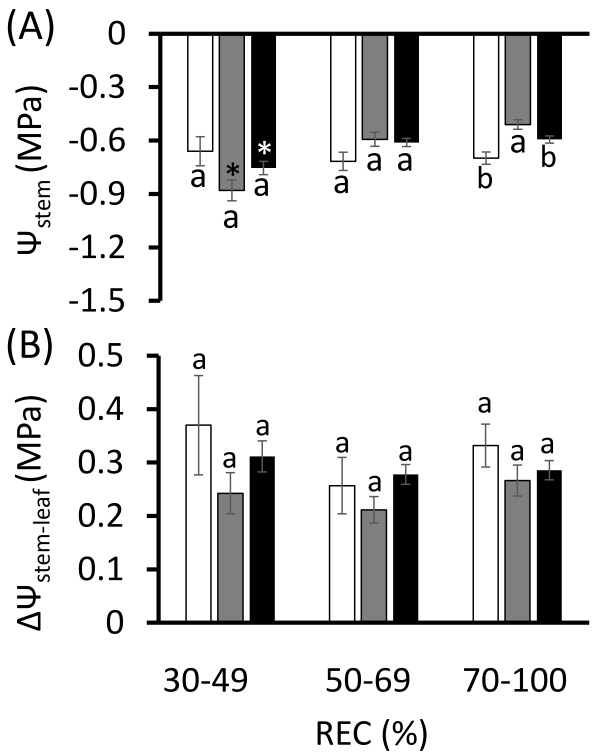

(A)
0
-0.3
-0.6
-0.9
-1.2
-1.5
Ψstem (MPa)
a
*
a
*
a
a
a
a
b
a
b
(B)
0.5
0.4
0.3
0.2
0.1
0
ΔΨstem-leaf (MPa)
a
a
a
a
a
a
a
a
a
30-49
50-69
70-100
REC (%)

Figure S1
